# Case Report: Rare pulmonary infection and cytomegalovirus retinitis revealed a case of lymphoma

**DOI:** 10.3389/fmed.2026.1732360

**Published:** 2026-02-13

**Authors:** Jinming Chen, Jie Yang, Yaxin Zhang

**Affiliations:** Department of Infectious Diseases, The Fifth Affiliated Hospital of Wenzhou Medical University, Lishui Central Hospital, Lishui, China

**Keywords:** cytomegalovirus retinitis, Epstein–Barr virus-positive diffuse large B-cell lymphoma, not otherwise specified (EBV+DLBCL-NOS), metagenomic next-generation sequencing (mNGS), *Penicillium digitatum*, Pulmonary infection, *Tropheryma whipplei*

## Abstract

**Background:**

Recurrent cytomegalovirus retinitis (CMVR) and rare opportunistic pulmonary infections may be the initial manifestations of underlying immunodeficiency caused by occult hematologic malignancy. Epstein–Barr virus-positive diffuse large B-cell lymphoma, not otherwise specified (EBV^+^DLBCL-NOS) is an aggressive lymphoma associated with immune dysfunction, predisposing patients to severe opportunistic infections, including CMVR. However, pulmonary co-infection with *Tropheryma whipplei* and *Penicillium digitatum* has not been previously described as a presenting feature of EBV^+^ DLBCL-NOS.

**Case:**

A 66-year-old male presented with blurred vision and was diagnosed with CMVR, with profoundly low CD4^+^ T-cell counts (102 cells/μL) and high cytomegalovirus (CMV) DNA levels in blood and aqueous humor. He initially responded to ganciclovir, but CMVR recurred five months later, accompanied by new pulmonary nodules. Despite negative conventional microbiological tests, metagenomic next-generation sequencing (mNGS) of bronchoalveolar lavage fluid identified co-infection with *Tropheryma whipplei* and *P. digitatum*. Broad-spectrum antimicrobial therapy led to partial clinical improvement, but pulmonary lesions persisted. PET-CT revealed hypermetabolic lung and lymph node lesions, and subsequent lung biopsy confirmed EBV^+^ DLBCL-NOS. The patient’s progressive immunodeficiency, recurrent CMVR, and refractory pulmonary infection were ultimately attributed to underlying lymphoma.

**Conclusion:**

This case highlights that severe, unexplained immunodeficiency with recurrent CMVR and rare opportunistic pulmonary infections should prompt a high index of suspicion for underlying hematologic malignancy. mNGS and PET-CT are critical tools in the diagnostic workup, but definitive diagnosis relies on histopathological confirmation. Early recognition of such presentations can prevent delays in diagnosing aggressive lymphomas.

## Introduction

Multiple opportunistic lung infections pose significant diagnostic challenges in immunocompromised patients, particularly when involving rare pathogen combinations. *Tropheryma whipplei*, a Gram-positive opportunistic bacterium, can cause pulmonary manifestations including multiple nodules and patchy infiltrates with rapid radiographic progression (1). *Penicillium digitatum*, a pathogen usually infects plants, has been reported in only three global cases of human pulmonary infection. All cases mainly occurred in individuals with low immune function and citrus fruit exposure (2, 3). Conventional microbiological cultures have limited utility for diagnosing these fastidious organisms, while metagenomic next-generation sequencing (mNGS) offers a valuable diagnostic alternative (4). When such rare infections fail to respond to targeted antimicrobial therapy, an underlying malignant process should be suspected. Epstein–Barr virus-positive diffuse large B-cell lymphoma, not otherwise specified (EBV^+^DLBCL-NOS), is an aggressive B-cell lymphoma that can present with insidious onset and extranodal involvement. Patients often exhibit immune dysfunction, predisposing them to opportunistic infections. Rare co-infections, including cerebral cryptococcosis and toxoplasmosis, have been documented in these patients. Here, we present a unique case in which recurrent cytomegalovirus retinitis (CMVR) and a mixed pulmonary infection with *Tropheryma whipplei* and *P. digitatum* were the initial manifestations of an underlying EBV^+^DLBCL-NOS. While CMVR is a well-documented complication in lymphoma patients (5), pulmonary co-infection with *Tropheryma whipplei* and *P. digitatum* remains exceptionally rare. This highlights the critical importance of maintaining a high index of suspicion for underlying malignancy in adults presenting with unexplained severe immunodeficiency and refractory, multifocal infections.

### Case

**Figure 1:**
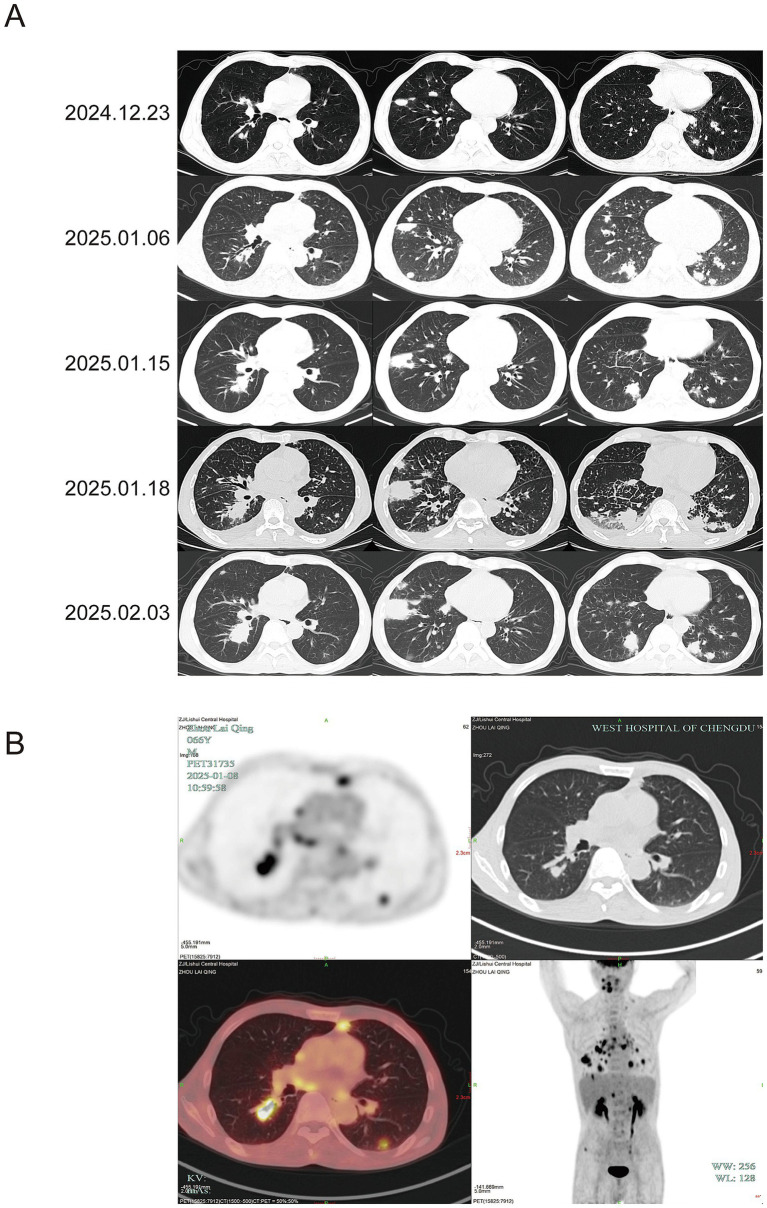
Description of chest CT and PET-CT during the treatment process. **(A)** Serial chest CT images from 23 December 2024 to 2 February 2025. Initial scan (23 December 2024) showed multiple bilateral nodules and patchy consolidations, suggestive of pneumonia. No treatment was initiated. Follow-up on 6 January 2025 indicated mild progression. In conjunction with mNGS, we first considered *Tropheryma whipplei* infection, leading to treatment with ceftriaxone and SMZ-TMP. By 15 January 2025, the lesions and high fever persisted. Therapy was switched to meropenem and doxycycline. On 18 January 2025, imaging revealed marked infiltration and moderate pleural effusion, accompanied by persistent high fever. Voriconazole was added against *P. digitatum*. By 2 February 2025, consolidations and effusion had resolved, but nodular lesions progressed. **(B)** On 6 January 2025, PET-CT image demonstrates multiple scattered spots, nodules, and patchy opacities were observed in both lungs. The largest lesion, located in the right lower lobe, measured approximately 34.9 × 20.1 mm with a maximum standardized uptake value (SUV_max_) of 11.3, highly suggestive of fungal infection. Additionally, foci were identified at the roots of the maxillary and mandibular teeth and in the right iliac bone region. Multiple lymph nodes were noted in the bilateral hilar, mediastinal, and left supraclavicular regions, as well as in the retroperitoneum. The corresponding SUV_max_ were 9.8, 4.3, 12.0, and 5.5, respectively.

A 66-year-old male was first evaluated at an outside hospital in June 2024 for fatigue, night sweats and blurred vision. Fundus examination showed a yellow-white retinal lesion with hemorrhage in the left eye. Routine blood tests revealed elevated C-reactive protein (CRP) (28.5 mg/L), leukocytopenia (2.6 × 10^9^/L), and lymphocytopenia (0.8 × 10^9^/L). Cytomegalovirus (CMV) DNA levels were markedly elevated in both blood (38,500 IU/mL) and aqueous humor (663,000 IU/mL). Chest CT was unremarkable. Lymphocyte subset analysis revealed severe immunodeficiency (CD4^+^ T cells: 102 cells/μL; CD8^+^T cells: 202 cells/μL; CD4^+^ T cells / CD8^+^ T cells ratio: 0.54). Bone marrow aspiration showed granulocytic hyperplasia without abnormal blasts. He was diagnosed with CMVR and treated with oral and intravitreal ganciclovir for five months. After CMV DNA in blood and aqueous humor became undetectable and CD4^+^ T cells increased to 114 cells/μL, therapy was discontinued in November 2024. Follow-up fundus examination at an external hospital’s outpatient department showed resolution of the left retinal hemorrhage. In December 2024, CMV DNA became detectable in both blood and aqueous humor again, and ganciclovir treatment was resumed (Supplementary Figure S5). Follow-up chest CT revealed nodular and patchy shadows in both lungs (Figure 1A), suggesting pulmonary infection. Hospitalization was recommended, but the patient declined.

**Figure 2:**
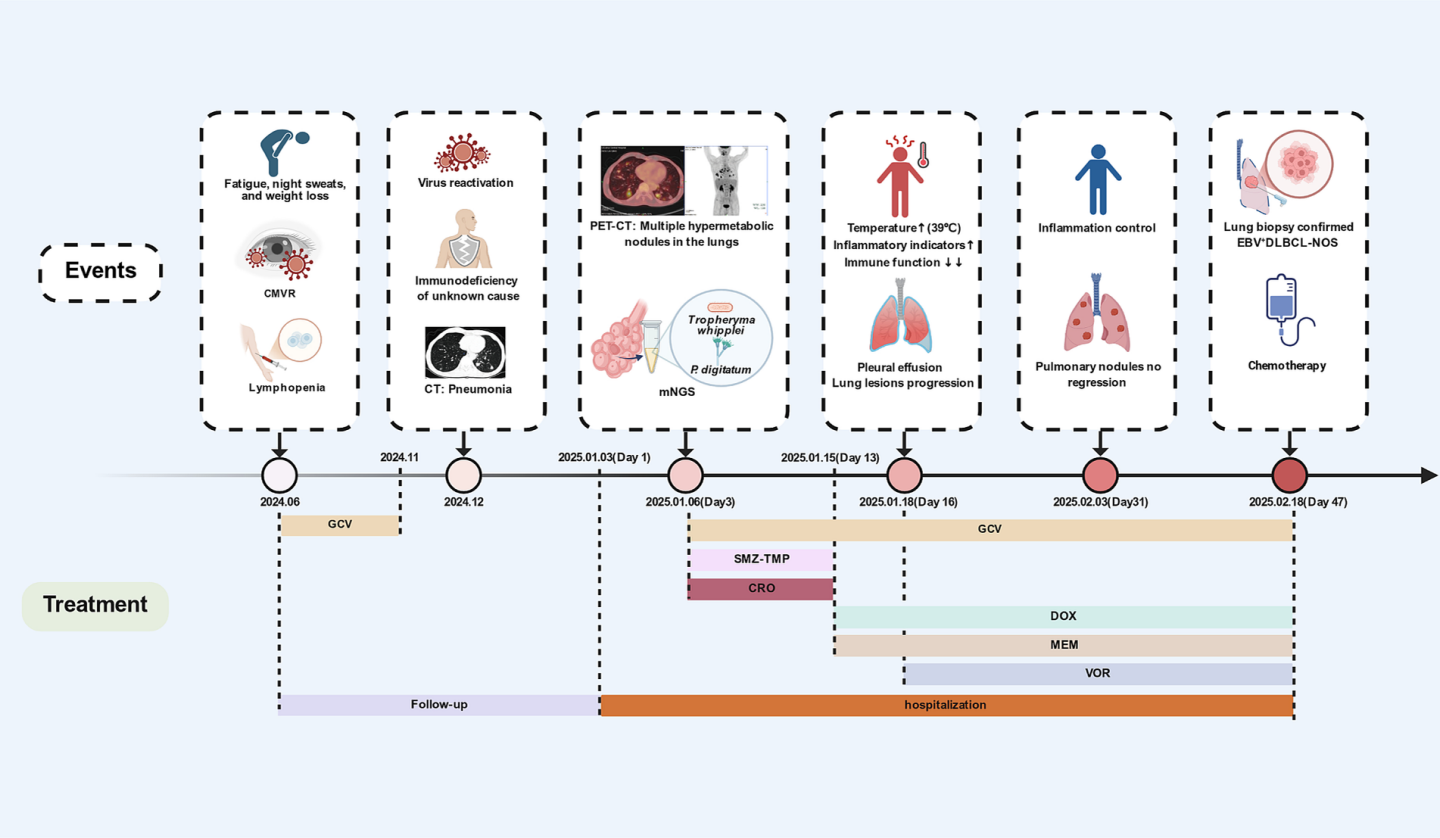
Case events and course of treatment. CMVR: cytomegalovirus retinitis; mNGS: metagenomic next-generation sequencing; EBV^+^DLBCL-NOS: Epstein–Barr virus-positive diffuse large B-cell lymphoma, not otherwise specified; SMZ-TMP: Sulfamethoxazole-trimethoprim. CRO: Ceftriaxone; DOX: Doxycycline; GCV: Ganciclovir; MEM: Meropenem; VOR: Voriconazole. Created with BioRender.com.

On January 3, 2025 (Day 1), the patient was admitted to our infectious disease department. He was afebrile and denied any history of pulmonary disease, malignancy, treatment with immune agents, travel to endemic regions, or animal contact, but he had a long-standing weight loss, night sweats and habit of consuming citrus fruits. Laboratory findings included elevated CRP (57.6 mg/L) and procalcitonin (PCT) (0.47 ng/mL), with further reduction in CD4^+^ T cells (64 cells/μL), CD8^+^ T cells (102 cells/μL), and a CD4^+^/CD8^+^ ratio of 0.62 (Table 1). The working diagnosis included pulmonary infection, CMVR, and immunodeficiency. Treatment with oral ganciclovir, thymalfasin, and immunoglobulin was initiated for immunomodulation. The underlying immunodeficiency remained undefined: HIV, antinuclear antibodies, and anti-interferon-*γ* autoantibodies were negative, and immunoglobulin levels were unremarkable. Therefore, whole-body PET-CT was performed to evaluate systemic conditions: multiple hypermetabolic pulmonary nodules and patches, findings highly suggestive of fungal infection (Figure 1B). Additionally, hypermetabolic lymph nodes were identified in the hilar, mediastinal, and supraclavicular regions (suggestive of reactive hyperplasia) and in the retroperitoneum (suspicious for hematologic malignancy). Similarly, the causative agent of pulmonary infection remained unclear: TB (T-SPOT, acid-fast bacilli stain, TB-DNA), fungal infections (galactomannan, (1,3)-*β*-D-glucan, cryptococcus), and Bronchoalveolar lavage fluid and sputum culture were all negative. BALF and blood were submitted for mNGS (Table 2). We initially considered pulmonary infection caused by *Tropheryma whipplei*. Empiric antimicrobial therapy with ceftriaxone and sulfamethoxazole was initiated on January 9 (Day 6). On January 12 (Day10), the patient developed high fever (39 °C) without chills or respiratory symptoms. Inflammatory markers remained elevated. On January 15(Day 13), chest CT showed progression of pulmonary infiltrates with enlarging consolidations and nodules (Figure 1A), alongside worsened leukopenia (2.3 × 10^9^/L) and lymphocytopenia (0.1 × 10^9^/L). Antibiotics were escalated to meropenem and doxycycline. A lung biopsy was proposed to rule out lymphoma but was refused by the patient, who requested transfer to a higher-level hospital (Figure 2).

**Table 1:** Patient clinical characteristics at admission.

Characteristics				
2025-01-03 (day 1)				
General data				
Age	66 years old			
Sex	Male			
Vital signs				
T	37.2°C			
RR	20 breaths/min			
BP	100/69 mmHg			
Ht	1.66 m			
Wt	57.5 kg			
BMI	20.87 kg/m^2^			
PS score	0			
Symptoms & physical examination				
Pulmonary auscultation	Wet rales			
B symptoms	Night sweats 5 kg weight loss (<6 month)			
Medical history				
Recent food	Citrus			
Allergy	Penicillin			
Disease	Hemorrhoidectomy			
Laboratory findings	2 months before admission	2025-01-03 (Day 1)	2025-01-18 (Day 16)	2025-02-18 (Day 47)
PCT (<0.05 ng/mL)	–	0.47	4.62	0.2
CRP (<8.0 mg/L)	–	50.75	70.05	10
ESR (0.0–15.0 mm/h)	–	32	–	–
WBC (3.5–9.5 × 10^9^/L)	2.4	2.3	4.02	2.85
N (1.8–6.3 × 10^9^/L)	1.8	1.9	3.69	1.24
LYM (1.1–3.2 × 10^9^/L)	0.3	0.2	0.17	1.39
Hb (130–175 g/L)	122	94	96	102
Plt (125–350 × 10^9^/L)	116	110	64	123
ALT (9–50 U/L)	13	26	31	40
AST (15–40 U/L)	19	18	20	20
TBIL (<23.0 μmol/L)	11.1	8.6	6.8	8
ALB (40–55 g/L)	43.8	29.6	27.6	34.4
GLB (20–40 g/L)	33.2	22.9	19.5	27
SF (21.8–274.7 ng/ml)	490.7	1093.6	–	–
PT (10.0–14.0 s)	–	12.3	–	13.5
Cr (57–111 μmol/L)	88	67	59	54
eGFR (>90 mL/min/1.73m^2^)	79	95.4	99.6	103.3
LDH (109–245 U/L)	–	212	–	346
CD4^+^ T cells (478–1,072 cells/μl)	114	64	34	–
CD8^+^ T cells (393–742 cells/μl)	188	102	47	–
NK cells	68	-	3	–
IgG (7.00–16.00 g/L)	19	12.5	–	14.6
IgA (0.70–4.0 g/L)	0.71	0.53	–	0.48
IgM (0.40–2.3 g/L)	1.24	0.73	–	0.51
IgE (<100 IU/mL)	8	10	–	–
HIV Ag/Ab (Neg)	–	Neg	–	–
EBV DNA (<200 IU/mL)	–	–	9,280	–

ALB: albumin; ALT: alanine aminotransferase; AST: aspartate aminotransferase; BP: blood pressure; BMI: body mass index; CRP: C-reactive protein; ESR: erythrocyte sedimentation rate; GLB: globulin; Hb: hemoglobin; Ht: height; IgA: immunoglobulin A; IgE: immunoglobulin E; IgG: immunoglobulin G; IgM: immunoglobulin M; LDH: lactate dehydrogenase; LYM: lymphocytes; N: Neutrophil; PCT: procalcitonin; PLT: platelets; PT: prothrombin time; RR: respiratory rate; T: temperature; TBIL: total bilirubin; WBC: white blood cells; Wt: weight.

**Table 2:** mNGS results of bronchoalveolar lavage fluid and blood.

Sample	BALF		Blood		
Category	*G^+^* Bacteria	Fungi	ds-DNA virus		
Species	*Tropheryma whipplei*	*P. digitatum*	*Human gammaherpesvirus 4* (EBV)	*Human herpesvirus 7*	*Human gammaherpesvirus 5* (CMV)
Detected sequence number	43,752	63	342	91	51
Relative abundance	44.26%	36.73%	3.97%	1.18%	0.43%

BALF: Bronchoalveolar lavage fluid; *G^+^*: Gram-positive; ds-DNA: double-stranded DNA; EBV: Epstein–Barr virus; CMV: Cytomegalovirus.

After transfer, the patient had persistent fever (38–39.5 °C) and developed chest tightness. On January 18 (Day 16), chest CT demonstrated progressive pulmonary lesions and pleural effusion (Figure 1A). Inflammatory markers increased further (CRP 70.05 mg/L, PCT 4.62 ng/mL) and the platelet (Plt) count was significantly lower (Plt 64 × 10^9^/L), suggesting severe infection. Thoracentesis and cytological examination of pleural fluid showed no abnormalities. The patient was immunodeficient and had poor infection control, so we considered concomitant *P. digitatum* infection and added voriconazole antifungal therapy. After 10 days (Day 26), the fever resolved, and inflammatory markers decreased (CRP 46.32 mg/L, PCT 1.02 ng/mL). Chest CT on February 2 (Day 31) revealed significant resolution of patchy pulmonary infiltrates and pleural effusion, but nodular lesions remained unchanged. (Figure 1A), with severe lymphocytopenia (0.07 × 10^9^/L). The patient then consented to lung biopsy. On February 18 (Day 47), the inflammatory markers were well-controlled (CRP 10 mg/L, PCT 0.2 ng/mL). Biopsy of the right lung mass revealed atypical lymphoid hyperplasia with necrosis and positive for EBV and CMV. Immunohistochemistry supported the diagnosis of EBV^+^ DLBCL-NOS (Supplementary Figures S1–S4). The patient was subsequently transferred to hematology for chemotherapy. The patient eventually died of multiorgan failure 10 months after chemotherapy due to systemic lymphoma metastasis (further details were not available). Finally, we monitored the longitudinal changes in white blood cell (WBC) counts, T lymphocyte subsets (CD4^+^ and CD8^+^ T cells), inflammatory markers (CRP and PCT), and CMV levels before diagnosis. (Supplementary Figure S5).

## Discussion

Rare pathogen pneumonia refers to opportunistic pulmonary infections caused by atypical, uncommon, or environmentally sourced microorganisms, commonly occurring in immunocompromised patients or those with underlying pulmonary diseases. These pathogens include bacteria (non-tuberculous mycobacteria, *Tropheryma* spp., *Chlamydia*), fungi (Aspergillus spp.*, Mucor* spp., *Talaromyces* spp., *Penicillium* spp., *Pneumocystis*), viruses (HHV, TTV) and other viruses (6, 7). Their clinical and radiographic presentations are often nonspecific. The diagnosis and treatment of rare pathogen pneumonia remain a therapeutic challenge in modern medicine.

Conventional diagnostic methods (standard culture, serology, and PCR), frequently fail to identify these pathogens due to their fastidious growth requirements, low microbial burden, or the sheer breadth of possible agents. The advent of mNGS has revolutionized the diagnosis of such infections (4). The incidence of such pneumonia is rising, paralleling the growing population of patients with compromised immunity due to hematologic malignancies, solid organ transplantation, long-term immunosuppressive therapy, or advanced HIV. In these individuals, impaired cellular and humoral defenses create a permissive environment for these unusual organisms to cause disease. CD4^+^ T cell count strongly predicts rare pathogen infection risk. a meta-analysis of BALF mNGS in 141 HIV patients showed increased rates of mixed and multiple pathogen infections when CD4^+^ T-cell counts were <200 cells/μL, with further increases observed when counts were <100 cells/μL (8). The management of these pneumonias is complex, and active identification of pathogens and immunodeficiency, anti-infection are the keys to treatment.

EBV^+^DLBCL-NOS is an aggressive B-cell lymphoma first described in 2003 by Oyama et al. in 22 elderly Japanese patients (9). The prognosis is poor, with a median overall survival of 36 months and a 5-year survival rate of 25% (10). EBV^+^DLBCL-NOS can involve rare or atypical anatomical sites, presenting with an occult onset and nonspecific manifestations. Documented cases in the literature include primary central nervous system involvement (11), gastrointestinal tract (12), trachea, and polyurethane-coated breast implants (13). The occurrence of these unusual sites indicates that the disease has a wide anatomical distribution, which poses challenges for early diagnosis. Lymphoma patients are at a significantly elevated risk for pulmonary infections, a leading cause of morbidity and mortality in this population. This susceptibility stems from a complex interplay of intrinsic immune dysfunction due to the underlying malignancy and extrinsic immunosuppression from cytotoxic chemotherapy, targeted agents, and immunomodulatory therapies. For endogenous immunosuppression, growing evidence links EBV^+^DLBCL-NOS to declining immune function, characterized by reduced CD4^+^ and CD8^+^ T cell counts (14, 15). This association may relate to specific EBV infection mechanisms.

*Tropheryma whipplei* is a Gram-positive, rod-shaped, obligate intracellular bacterium belonging to the Actinobacteria phylum. While gastrointestinal tract and joints involvement is classic, pulmonary infection caused by *Tropheryma whipplei* is increasingly recognized, particularly in immunocompromised individuals or those with structural lung disease. At present, only 4 cases of lymphoma patients with pulmonary infection have been reported (16–19) (Table 3). Their clinical features are nonspecific, while radiological findings such as ground-glass opacities, interstitial infiltrates, and pulmonary nodules mimic lymphoma relapse, drug-induced pneumonitis, or other opportunistic infections. Therefore, the diagnosis of *Tropheryma whipplei* pneumonia in lymphoma patients remains exceptionally challenging. Periodic acid–Schiff (PAS) staining lacks sensitivity and specificity, targeted PCR may miss atypical strains, and culture is notoriously difficult. In immunosuppressed individuals, serological responses are often blunted. mNGS is the key to diagnosis.

**Table 3:** Literature review by *Tropheryma whipplei* and *P. digitatum*.

Year	Age/Sex	Symptoms	Medical History	Underlying Diseases	CT Features	Diagnostic Tests	Treatment	Prognosis
1. Lymphoma complicated with pulmonary infection of Tropheryma whipplei has been reported in the literature.								
2024	69/F (16)	Weight loss	Immunosuppressive agents, glucocorticoids, chemotherapy drugs	MALT	A consolidation in the right lower pulmonary lobe.	Lung Tissue mNGS	MFX CRO	Improved
2025	39/F (17)	Fever Cough	Immunosuppressive agents	2-year history of Lymphoma	Scattered inflammatory lesions, interstitial changes and ground-glass nodules.	BALF mNGS	MEM DOX SMZ	Improved
2025	61/M (18)	Dry cough	Immunosuppressive agents, glucocorticoids, chemotherapy drugs	B-cell Malignant Lymphoma	Bilateral ground-glass opacities.	BALF mNGS	CRO SMZ-TMP	Improved
2025	61/M (19)	Weight loss Nausea Vomiting	None	Bronchus-associated Lymphoid Tissue Lymphoma	6 cm suspicious left hilar mass.	PCR	CRO SMZ	Improved
2. Pulmonary infection by *P. digitatum* was reported in the literature.								
2013	78/M (3)	Physical examination	Citrus contact glucocorticoids	Asthma Emphysema	A fungus ball with 4 cm thin-walled cavity.	Sputum culture	ITZ CAS VOR AmB FLU	Deceased
2022	20/F (2)	Fever	Glucocorticoids	COVID-19	Consolidation of ground glass opacities and bilateral nodular opacities in the upper lobes.	BALF mNGS and culture	ITZ	Improved
2024	66/M (31)	Fever Cough	Citrus contact	COVID-19 Emphysema	Right lower lobe inflammatory lesions and local consolidation with small amount of pleural effusion.	BALF mNGS	ITZ	Improved

AmB: Amphotericin B; CAS: Caspofungin; CRO: Ceftriaxone; DOX: Doxycycline; FLU: Fluconazole; ITZ: Itraconazole; SMZ: Sulfamethoxazole; SMZ-TMP: Sulfamethoxazole-Trimethoprim; VOR: Voriconazole; MFX: Moxifloxacin; MEM: Meropenem; F: Female M: Male; MALT: Mucosa-Associated Lymphoid Tissue.

Initially, the patient presented only with cough and pulmonary nodules and consolidations, which were attributed to *Tropheryma whipplei* infection based on the BALF mNGS results. Antibiotics generally effective against *Tropheryma whipplei* include penicillin, ceftriaxone, meropenem, streptomycin, tetracycline, SMZ-TMP, doxycycline, and hydroxychloroquine (20). Current main recommendations are ceftriaxone or meropenem for 14 days, followed by SMZ-TMP for one year (21). This patient had immunodeficiency and CMVR. Monotherapy might be ineffective, and hydroxychloroquine should to be avoided due to potential retinal toxicity. Therefore, initial treatment with ceftriaxone combined with SMZ-TMP was started. However, high fever persisted during treatment, and pulmonary progression was observed, potentially related to SMZ-TMP resistance (22). Therapy was then changed to meropenem combined with doxycycline, but efficacy remained poor and pleural effusion and progressive lung exudative lesions were present.

*Penicillium* spp. are ubiquitous environmental molds, and human infections are rare. Although often regarded as contaminants in clinical settings, invasive infections should be considered in immunocompromised hosts (23). Pulmonary infections caused by *P. chrysogenum* (24), *P. citrinum* (25), *P. janthinellum* (26), *P. digitatum* (2), *P. oxalicum* (27), *P. notatum* (28), and *P. capsulatum* (29) have been reported, with manifestations including disseminated parenchymal infiltration, lobar pneumonia, bronchopneumonia, fungal ball, and pleural effusion (30). To date, only 3 global cases of *P. digitatum* infection have been documented, with risk factors including corticosteroid use, COVID-19, and exposure to citrus fruits (2, 3, 31).

In this case, the patient exhibited progressive pneumonia despite broad-spectrum antibiotic therapy, in the context of underlying immunodeficiency. A detailed history revealed long-term citrus consumption. mNGS of BALF detected *P. digitatum*-specific sequences with high relative abundance (36.73%), supporting the diagnosis of co-infection. Although both serum galactomannan and (1,3)-*β*-D-glucan assays were negative, this is consistent with prior reports by Isabel Iturrieta-González et al. (2), in which *P. digitatum* infection also yielded negative biomarkers The limited diagnostic value of these serological tests in this context is likely due to the high specificity of galactomannan for Aspergillus spp., as well as severely impaired host immune responses in profoundly immunocompromised individuals, resulting in minimal release of fungal components into the bloodstream.

Currently, there is no standardized guideline for the treatment of *P. digitatum* infection. However, case reports by Isabel Iturrieta-González et al. (2) and Xiaojuan Shi et al. (31) demonstrated susceptibility to voriconazole. In addition, an *in vitro* antifungal susceptibility study of various *Penicillium* isolates showed favorable activity of triazoles and echinocandins (30). Unfortunately, fungal culture was negative in this case, which is not uncommon given the fastidious growth requirements of *Penicillium* spp. Empirical voriconazole therapy was initiated and resulted in significant clinical improvement.

The pulmonary lesions were complex. Initial presentation was characterized by marked elevation of inflammatory markers and rapid progression of pulmonary infiltrates, consistent with active co-infection involving *Tropheryma whipplei* and *P. digitatum*. Following treatment with meropenem, doxycycline, and voriconazole, inflammatory markers and exudative lesions improved, though persistent nodular opacities remained. Although PET-CT is crucial for evaluating systemic tumor burden, its utility is limited in differentiating between pulmonary atypical pathogen infection lesions and lymphoma, as infection by *Tropheryma whipple* or fungi can exhibit high SUV_max_ (32, 33). Therefore, early percutaneous lung biopsy was necessary. Unfortunately, the patient initially refused, which may have partially delayed interpretation of the complex condition. Eventually, percutaneous lung nodule biopsy confirmed lymphoma, explaining the primary cause of immunodeficiency and atypical pathogen infection. Ultimately, percutaneous lung biopsy confirmed the diagnosis of lymphoma, which explained the basis of the patient’s immunodeficiency and susceptibility to opportunistic infections. We infer that the early phase was predominantly infectious with superimposed lymphomatous involvement, evolving into a lymphoma-dominant proliferative process during follow-up.

Furthermore, while CMVR is most associated with HIV infection, it can also occur in patients with hematologic malignancies (5) or solid organ tumors (34). According to HIV opportunistic infection guidelines, discontinuing therapy is safe when CMVR is quiescent and CD4^+^ T cells remain >100 cells/μL for 3–6 months (35). The patient received 5 months of oral ganciclovir plus intravitreal injections, with subsequent undetectable CMV DNA in blood and aqueous humor and CD4^+^ T cells stably >100 cells/μL for 3 months (Supplementary Figures S5B–D). We consider the prior treatment to have been appropriate and sufficient. CMV DNA load is inversely correlated with CD4^+^ T cell counts, a relationship observed in this patient. Despite prolonged and appropriate ganciclovir therapy, CMV DNA levels surged as CD4^+^ counts progressively declined, consistent with lymphoma-associated immunosuppression leading to CMV reactivation (36), suggesting occult progression of lymphoma. This underscores the limited efficacy of antiviral monotherapy in the setting of profound immunosuppression, highlighting the need for immune reconstitution alongside targeted antiviral treatment.

## Conclusion

This case illustrates a rare presentation of EBV^+^DLBCL-NOS, initially manifesting as CMVR and a complex pulmonary co-infection with *Tropheryma whipplei* and *P. digitatum* in a patient with severe, unexplained immunodeficiency. The diagnostic journey underscores several critical lessons. First, mNGS proved invaluable for identifying fastidious pathogens that eluded conventional microbiology, enabling targeted antimicrobial therapy. However, persistent radiographic progression despite appropriate treatment warranted further investigation. Second, PET-CT imaging, while helpful in assessing systemic disease burden, has limited specificity in distinguishing between infectious and malignant pulmonary processes, as both can exhibit high metabolic activity. Third, and most importantly, refractory, multifocal opportunistic infections in adults with profound immunosuppression, particularly when not attributable to HIV/AIDS or iatrogenic causes, should prompt urgent evaluation for an underlying occult hematologic malignancy. The definitive diagnosis ultimately required histopathological confirmation. We advocate early consideration of tissue biopsy when clinical suspicion persists despite antimicrobial therapy, to prevent critical delays in initiating appropriate oncologic management. This case reinforces the complex interplay between malignancy-associated immunosuppression and opportunistic pathogens, and highlights the necessity of a multidisciplinary approach in diagnosing and managing such challenging clinical presentations.

## Data Availability

The raw data supporting the conclusions of this article will be made available by the authors, without undue reservation.
