# Supplementary material for: Case Report: Rare pulmonary infection and cytomegalovirus retinitis revealed a case of lymphoma
Source: Front Med (Lausanne). 2026 Feb 13;13:1732360. doi: 10.3389/fmed.2026.1732360 (PMC12945748; doi:10.3389/fmed.2026.1732360)
Supplement: Supplementary file 1 [file Table_1.docx]

**Supplementary**


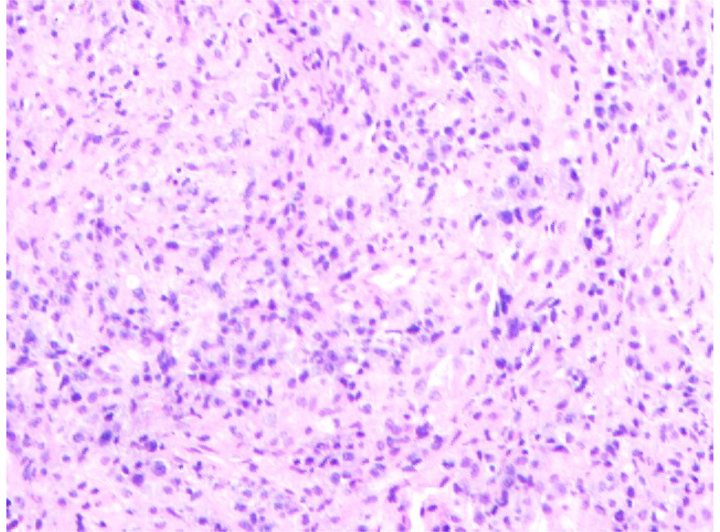


**S1: Representative histopathology of the patient's lung biopsy (H&E staining):** The pulmonary interstitium demonstrates lymphocyte hyperplasia, with a subset of lymphocytes exhibiting approximately twofold enlargement in volume, accompanied by irregular nuclear contours and hyperchromatic nuclei.

**S2: Immunohistochemical results revealed the following profile:** CD3 (scattered T-cells+), CD20 (scattered B-cells+), Ki-67 (60%+), CD30 (scattered+), CD21 (-), EBER (+), CK(pan) (-), TTF-1 (-), P40 (-), CD34 (-), S-100 (-), MUM1 (+), CMV2 (scattered+), CD56 (-), and CD5 (scattered T-cells+), CD79a(B+)，CD138(-)，CD38(+)，Kappa(K)(-)，Lambda(λ)(-).

**S3: Molecular testing:** IG gene rearrangement (+).

**S4: Pathological Diagnosis:** (Right lung mass biopsy) Atypical lymphoid hyperplasia with EBV and CMV infection and necrosis. Combined with the immunohistochemical profile, the findings are consistent with EBV^+^ DLBCL-NOS.


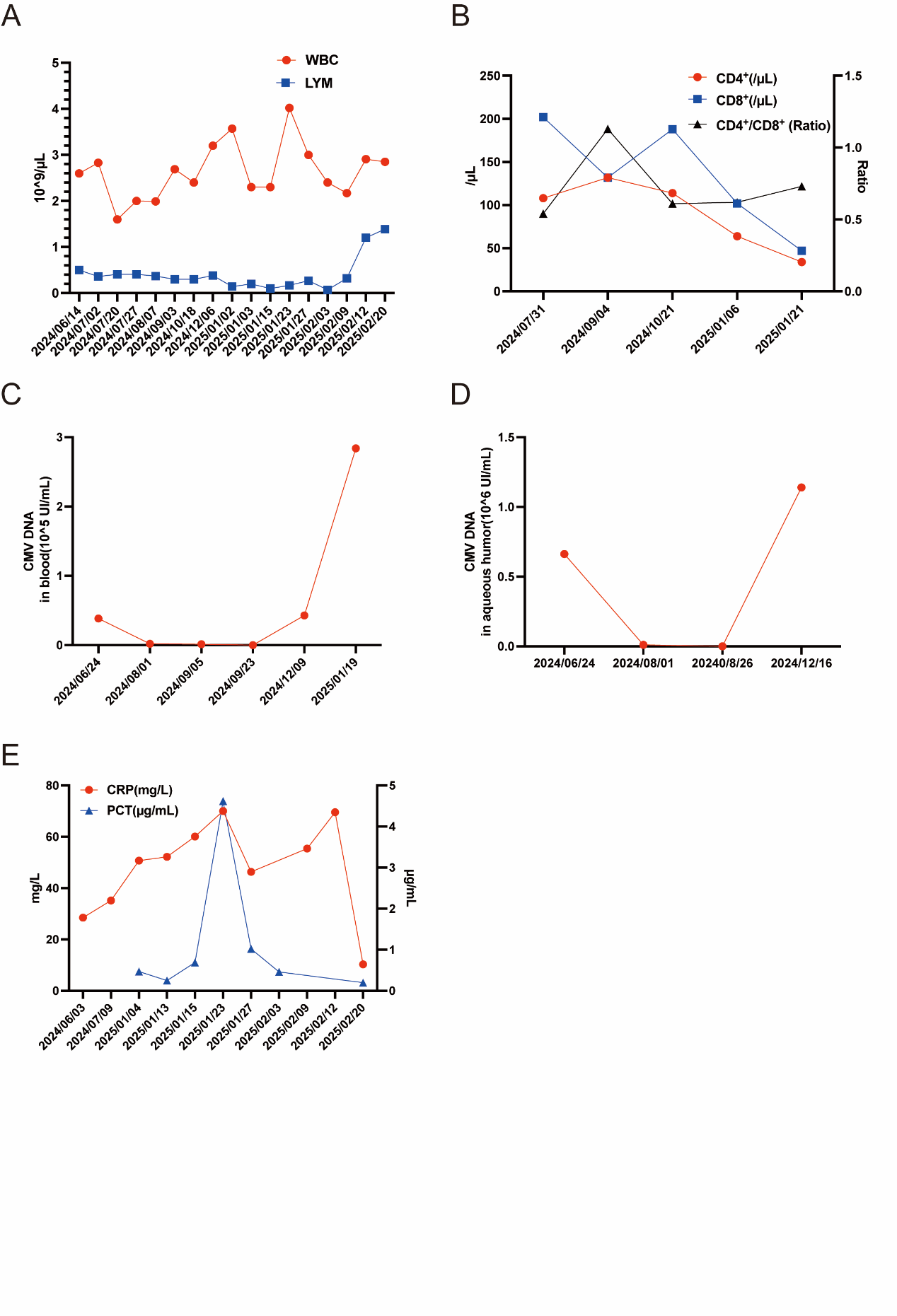


**S5: Relevant laboratory test throughout the disease course. (A)** Laboratory test results of white blood cells and lymphocyte. **(B)** Laboratory test results of lymphocyte subsets (CD4^+^ and CD8^+^ T cells). **(C-D)** Cytomegalovirus levels in blood and aqueous humor. **(E)** Laboratory test results of inflammatory markers (C-reactive protein and Procalcitonin). WBC: white blood cells; LYM: lymphocytes; CRP: C-reactive protein; PCT: Procalcitonin; CD4: CD4^+^ T cells; CD8: CD8^+^ T cells.
